# Supplementary material for: COZOID: contact zone identifier for visual analysis of protein-protein interactions
Source: BMC Bioinformatics. 2018 Apr 6;19:125. doi: 10.1186/s12859-018-2113-6 (PMC5889581; doi:10.1186/s12859-018-2113-6)
Supplement: Supplementary file 2 — Software build. Executable binary file of the software tool. (ZIP 118,784 kb) [file 12859_2018_2113_MOESM2_ESM.zip › cozoid/java/docs/beansbinding-1.2.1-doc.zip!/org/jdesktop/swingbinding/SwingBindings.html]

SwingBindings (Beans Binding)


---


|  |  |  |
| --- | --- | --- |
| **Overview** \| **Package** \| **Class** \| **Use** \| **Tree** \| **Deprecated** \| **Index** \| **Help** | |  |
| **PREV CLASS**   NEXT CLASS | **FRAMES**    **NO FRAMES**     **All Classes** |  |
| SUMMARY: NESTED \| FIELD \| CONSTR \| METHOD | DETAIL: FIELD \| CONSTR \| METHOD |  |


---


## org.jdesktop.swingbinding Class SwingBindings

```
java.lang.Object
  org.jdesktop.swingbinding.SwingBindings
```

---

``` public class SwingBindings extends java.lang.Object ```

A factory class for creating instances of the custom Swing `Binding`
implementations provided by this package. See the
package summary for full details on
binding to Swing components.

---

| **Method Summary** | |
| --- | --- |
| `static <E> JComboBoxBinding<E,java.util.List<E>,javax.swing.JComboBox>` | `createJComboBoxBinding(AutoBinding.UpdateStrategy strategy, java.util.List<E> sourceList, javax.swing.JComboBox targetJComboBox)`             Creates a `JComboBoxBinding` from direct references to a `List` and `JComboBox`. |
| `static <E> JComboBoxBinding<E,java.util.List<E>,javax.swing.JComboBox>` | `createJComboBoxBinding(AutoBinding.UpdateStrategy strategy, java.util.List<E> sourceList, javax.swing.JComboBox targetJComboBox, java.lang.String name)`             Creates a named `JComboBoxBinding` from direct references to a `List` and `JComboBox`. |
| `static <E,TS> JComboBoxBinding<E,java.util.List<E>,TS>` | `createJComboBoxBinding(AutoBinding.UpdateStrategy strategy, java.util.List<E> sourceList, TS targetObject, Property<TS,? extends javax.swing.JComboBox> targetJComboBoxProperty)`             Creates a `JComboBoxBinding` from a direct reference to a `List` and an object and property that resolves to a `JComboBox`. |
| `static <E,TS> JComboBoxBinding<E,java.util.List<E>,TS>` | `createJComboBoxBinding(AutoBinding.UpdateStrategy strategy, java.util.List<E> sourceList, TS targetObject, Property<TS,? extends javax.swing.JComboBox> targetJComboBoxProperty, java.lang.String name)`             Creates a named `JComboBoxBinding` from a direct reference to a `List` and an object and property that resolves to a `JComboBox`. |
| `static <E,SS> JComboBoxBinding<E,SS,javax.swing.JComboBox>` | `createJComboBoxBinding(AutoBinding.UpdateStrategy strategy, SS sourceObject, Property<SS,java.util.List<E>> sourceListProperty, javax.swing.JComboBox targetJComboBox)`             Creates a `JComboBoxBinding` from an object and property that resolves to a `List` and a direct reference to a `JComboBox`. |
| `static <E,SS> JComboBoxBinding<E,SS,javax.swing.JComboBox>` | `createJComboBoxBinding(AutoBinding.UpdateStrategy strategy, SS sourceObject, Property<SS,java.util.List<E>> sourceListProperty, javax.swing.JComboBox targetJComboBox, java.lang.String name)`             Creates a named `JComboBoxBinding` from an object and property that resolves to a `List` and a direct reference to a `JComboBox`. |
| `static <E,SS,TS> JComboBoxBinding<E,SS,TS>` | `createJComboBoxBinding(AutoBinding.UpdateStrategy strategy, SS sourceObject, Property<SS,java.util.List<E>> sourceListProperty, TS targetObject, Property<TS,? extends javax.swing.JComboBox> targetJComboBoxProperty)`             Creates a `JComboBoxBinding` from an object and property that resolves to a `List` and an object and property that resolves to a `JComboBox`. |
| `static <E,SS,TS> JComboBoxBinding<E,SS,TS>` | `createJComboBoxBinding(AutoBinding.UpdateStrategy strategy, SS sourceObject, Property<SS,java.util.List<E>> sourceListProperty, TS targetObject, Property<TS,? extends javax.swing.JComboBox> targetJComboBoxProperty, java.lang.String name)`             Creates a named `JComboBoxBinding` from an object and property that resolves to a `List` and an object and property that resolves to a `JComboBox`. |
| `static <E> JListBinding<E,java.util.List<E>,javax.swing.JList>` | `createJListBinding(AutoBinding.UpdateStrategy strategy, java.util.List<E> sourceList, javax.swing.JList targetJList)`             Creates a `JListBinding` from direct references to a `List` and `JList`. |
| `static <E> JListBinding<E,java.util.List<E>,javax.swing.JList>` | `createJListBinding(AutoBinding.UpdateStrategy strategy, java.util.List<E> sourceList, javax.swing.JList targetJList, java.lang.String name)`             Creates a named `JListBinding` from direct references to a `List` and `JList`. |
| `static <E,TS> JListBinding<E,java.util.List<E>,TS>` | `createJListBinding(AutoBinding.UpdateStrategy strategy, java.util.List<E> sourceList, TS targetObject, Property<TS,? extends javax.swing.JList> targetJListProperty)`             Creates a `JListBinding` from a direct reference to a `List` and an object and property that resolves to a `JList`. |
| `static <E,TS> JListBinding<E,java.util.List<E>,TS>` | `createJListBinding(AutoBinding.UpdateStrategy strategy, java.util.List<E> sourceList, TS targetObject, Property<TS,? extends javax.swing.JList> targetJListProperty, java.lang.String name)`             Creates a named `JListBinding` from a direct reference to a `List` and an object and property that resolves to a `JList`. |
| `static <E,SS> JListBinding<E,SS,javax.swing.JList>` | `createJListBinding(AutoBinding.UpdateStrategy strategy, SS sourceObject, Property<SS,java.util.List<E>> sourceListProperty, javax.swing.JList targetJList)`             Creates a `JListBinding` from an object and property that resolves to a `List` and a direct reference to a `JList`. |
| `static <E,SS> JListBinding<E,SS,javax.swing.JList>` | `createJListBinding(AutoBinding.UpdateStrategy strategy, SS sourceObject, Property<SS,java.util.List<E>> sourceListProperty, javax.swing.JList targetJList, java.lang.String name)`             Creates a named `JListBinding` from an object and property that resolves to a `List` and a direct reference to a `JList`. |
| `static <E,SS,TS> JListBinding<E,SS,TS>` | `createJListBinding(AutoBinding.UpdateStrategy strategy, SS sourceObject, Property<SS,java.util.List<E>> sourceListProperty, TS targetObject, Property<TS,? extends javax.swing.JList> targetJListProperty)`             Creates a `JListBinding` from an object and property that resolves to a `List` and an object and property that resolves to a `JList`. |
| `static <E,SS,TS> JListBinding<E,SS,TS>` | `createJListBinding(AutoBinding.UpdateStrategy strategy, SS sourceObject, Property<SS,java.util.List<E>> sourceListProperty, TS targetObject, Property<TS,? extends javax.swing.JList> targetJListProperty, java.lang.String name)`             Creates a named `JListBinding` from an object and property that resolves to a `List` and an object and property that resolves to a `JList`. |
| `static <E> JTableBinding<E,java.util.List<E>,javax.swing.JTable>` | `createJTableBinding(AutoBinding.UpdateStrategy strategy, java.util.List<E> sourceList, javax.swing.JTable targetJTable)`             Creates a `JTableBinding` from direct references to a `List` and `JTable`. |
| `static <E> JTableBinding<E,java.util.List<E>,javax.swing.JTable>` | `createJTableBinding(AutoBinding.UpdateStrategy strategy, java.util.List<E> sourceList, javax.swing.JTable targetJTable, java.lang.String name)`             Creates a named `JTableBinding` from direct references to a `List` and `JTable`. |
| `static <E,TS> JTableBinding<E,java.util.List<E>,TS>` | `createJTableBinding(AutoBinding.UpdateStrategy strategy, java.util.List<E> sourceList, TS targetObject, Property<TS,? extends javax.swing.JTable> targetJTableProperty)`             Creates a `JTableBinding` from a direct reference to a `List` and an object and property that resolves to a `JTable`. |
| `static <E,TS> JTableBinding<E,java.util.List<E>,TS>` | `createJTableBinding(AutoBinding.UpdateStrategy strategy, java.util.List<E> sourceList, TS targetObject, Property<TS,? extends javax.swing.JTable> targetJTableProperty, java.lang.String name)`             Creates a named `JTableBinding` from a direct reference to a `List` and an object and property that resolves to a `JTable`. |
| `static <E,SS> JTableBinding<E,SS,javax.swing.JTable>` | `createJTableBinding(AutoBinding.UpdateStrategy strategy, SS sourceObject, Property<SS,java.util.List<E>> sourceListProperty, javax.swing.JTable targetJTable)`             Creates a `JTableBinding` from an object and property that resolves to a `List` and a direct reference to a `JTable`. |
| `static <E,SS> JTableBinding<E,SS,javax.swing.JTable>` | `createJTableBinding(AutoBinding.UpdateStrategy strategy, SS sourceObject, Property<SS,java.util.List<E>> sourceListProperty, javax.swing.JTable targetJTable, java.lang.String name)`             Creates a named `JTableBinding` from an object and property that resolves to a `List` and a direct reference to a `JTable`. |
| `static <E,SS,TS> JTableBinding<E,SS,TS>` | `createJTableBinding(AutoBinding.UpdateStrategy strategy, SS sourceObject, Property<SS,java.util.List<E>> sourceListProperty, TS targetObject, Property<TS,? extends javax.swing.JTable> targetJTableProperty)`             Creates a `JTableBinding` from an object and property that resolves to a `List` and an object and property that resolves to a `JTable`. |
| `static <E,SS,TS> JTableBinding<E,SS,TS>` | `createJTableBinding(AutoBinding.UpdateStrategy strategy, SS sourceObject, Property<SS,java.util.List<E>> sourceListProperty, TS targetObject, Property<TS,? extends javax.swing.JTable> targetJTableProperty, java.lang.String name)`             Creates a named `JTableBinding` from an object and property that resolves to a `List` and an object and property that resolves to a `JTable`. |

**Methods inherited from class java.lang.Object**

`clone, equals, finalize, getClass, hashCode, notify, notifyAll, toString, wait, wait, wait`

**Method Detail**

### createJListBinding

```
public static <E> JListBinding<E,java.util.List<E>,javax.swing.JList> createJListBinding(AutoBinding.UpdateStrategy strategy,
                                                                                         java.util.List<E> sourceList,
                                                                                         javax.swing.JList targetJList)
```

:   Creates a `JListBinding` from direct references to a `List` and `JList`.

    :   **Parameters:**: `strategy` - the update strategy: `sourceList` - the source `List`: `targetJList` - the target `JList` **Returns:**: the `JTableBinding`

---


### createJListBinding

```
public static <E> JListBinding<E,java.util.List<E>,javax.swing.JList> createJListBinding(AutoBinding.UpdateStrategy strategy,
                                                                                         java.util.List<E> sourceList,
                                                                                         javax.swing.JList targetJList,
                                                                                         java.lang.String name)
```

:   Creates a named `JListBinding` from direct references to a `List` and `JList`.

    :   **Parameters:**: `strategy` - the update strategy: `sourceList` - the source `List`: `targetJList` - the target `JList` **Returns:**: the `JListBinding`

---


### createJListBinding

```
public static <E,SS> JListBinding<E,SS,javax.swing.JList> createJListBinding(AutoBinding.UpdateStrategy strategy,
                                                                             SS sourceObject,
                                                                             Property<SS,java.util.List<E>> sourceListProperty,
                                                                             javax.swing.JList targetJList)
```

:   Creates a `JListBinding` from an object and property that resolves to a `List` and a direct reference to a `JList`.

    :   **Parameters:**: `strategy` - the update strategy: `sourceObject` - the source object: `sourceListProperty` - a property on the source object that resolves to a `List`: `targetJList` - the target `JList` **Returns:**: the `JListBinding` **Throws:**: `java.lang.IllegalArgumentException` - if `sourceListProperty` is `null`

---


### createJListBinding

```
public static <E,SS> JListBinding<E,SS,javax.swing.JList> createJListBinding(AutoBinding.UpdateStrategy strategy,
                                                                             SS sourceObject,
                                                                             Property<SS,java.util.List<E>> sourceListProperty,
                                                                             javax.swing.JList targetJList,
                                                                             java.lang.String name)
```

:   Creates a named `JListBinding` from an object and property that resolves to a `List` and a direct reference to a `JList`.

    :   **Parameters:**: `strategy` - the update strategy: `sourceObject` - the source object: `sourceListProperty` - a property on the source object that resolves to a `List`: `targetJList` - the target `JList` **Returns:**: the `JListBinding` **Throws:**: `java.lang.IllegalArgumentException` - if `sourceListProperty` is `null`

---


### createJListBinding

```
public static <E,TS> JListBinding<E,java.util.List<E>,TS> createJListBinding(AutoBinding.UpdateStrategy strategy,
                                                                             java.util.List<E> sourceList,
                                                                             TS targetObject,
                                                                             Property<TS,? extends javax.swing.JList> targetJListProperty)
```

:   Creates a `JListBinding` from a direct reference to a `List` and an object and property that resolves to a `JList`.

    :   **Parameters:**: `strategy` - the update strategy: `sourceList` - the source `List`: `targetObject` - the target object: `targetJListProperty` - a property on the target object that resolves to a `JList` **Returns:**: the `JListBinding` **Throws:**: `java.lang.IllegalArgumentException` - if `targetJListProperty` is `null`

---


### createJListBinding

```
public static <E,TS> JListBinding<E,java.util.List<E>,TS> createJListBinding(AutoBinding.UpdateStrategy strategy,
                                                                             java.util.List<E> sourceList,
                                                                             TS targetObject,
                                                                             Property<TS,? extends javax.swing.JList> targetJListProperty,
                                                                             java.lang.String name)
```

:   Creates a named `JListBinding` from a direct reference to a `List` and an object and property that resolves to a `JList`.

    :   **Parameters:**: `strategy` - the update strategy: `sourceList` - the source `List`: `targetObject` - the target object: `targetJListProperty` - a property on the target object that resolves to a `JList` **Returns:**: the `JListBinding` **Throws:**: `java.lang.IllegalArgumentException` - if `targetJListProperty` is `null`

---


### createJListBinding

```
public static <E,SS,TS> JListBinding<E,SS,TS> createJListBinding(AutoBinding.UpdateStrategy strategy,
                                                                 SS sourceObject,
                                                                 Property<SS,java.util.List<E>> sourceListProperty,
                                                                 TS targetObject,
                                                                 Property<TS,? extends javax.swing.JList> targetJListProperty)
```

:   Creates a `JListBinding` from an object and property that resolves to a `List` and an object and property that resolves to a `JList`.

    :   **Parameters:**: `strategy` - the update strategy: `sourceObject` - the source object: `sourceListProperty` - a property on the source object that resolves to a `List`: `targetObject` - the target object: `targetJListProperty` - a property on the target object that resolves to a `JList` **Returns:**: the `JListBinding` **Throws:**: `java.lang.IllegalArgumentException` - if `sourceListProperty` or `targetJListProperty` is `null`

---


### createJListBinding

```
public static <E,SS,TS> JListBinding<E,SS,TS> createJListBinding(AutoBinding.UpdateStrategy strategy,
                                                                 SS sourceObject,
                                                                 Property<SS,java.util.List<E>> sourceListProperty,
                                                                 TS targetObject,
                                                                 Property<TS,? extends javax.swing.JList> targetJListProperty,
                                                                 java.lang.String name)
```

:   Creates a named `JListBinding` from an object and property that resolves to a `List` and an object and property that resolves to a `JList`.

    :   **Parameters:**: `strategy` - the update strategy: `sourceObject` - the source object: `sourceListProperty` - a property on the source object that resolves to a `List`: `targetObject` - the target object: `targetJListProperty` - a property on the target object that resolves to a `JList` **Returns:**: the `JListBinding` **Throws:**: `java.lang.IllegalArgumentException` - if `sourceListProperty` or `targetJListProperty` is `null`

---


### createJTableBinding

```
public static <E> JTableBinding<E,java.util.List<E>,javax.swing.JTable> createJTableBinding(AutoBinding.UpdateStrategy strategy,
                                                                                            java.util.List<E> sourceList,
                                                                                            javax.swing.JTable targetJTable)
```

:   Creates a `JTableBinding` from direct references to a `List` and `JTable`.

    :   **Parameters:**: `strategy` - the update strategy: `sourceList` - the source `List`: `targetJTable` - the target `JTable` **Returns:**: the `JTableBinding`

---


### createJTableBinding

```
public static <E> JTableBinding<E,java.util.List<E>,javax.swing.JTable> createJTableBinding(AutoBinding.UpdateStrategy strategy,
                                                                                            java.util.List<E> sourceList,
                                                                                            javax.swing.JTable targetJTable,
                                                                                            java.lang.String name)
```

:   Creates a named `JTableBinding` from direct references to a `List` and `JTable`.

    :   **Parameters:**: `strategy` - the update strategy: `sourceList` - the source `List`: `targetJTable` - the target `JTable` **Returns:**: the `JTableBinding`

---


### createJTableBinding

```
public static <E,SS> JTableBinding<E,SS,javax.swing.JTable> createJTableBinding(AutoBinding.UpdateStrategy strategy,
                                                                                SS sourceObject,
                                                                                Property<SS,java.util.List<E>> sourceListProperty,
                                                                                javax.swing.JTable targetJTable)
```

:   Creates a `JTableBinding` from an object and property that resolves to a `List` and a direct reference to a `JTable`.

    :   **Parameters:**: `strategy` - the update strategy: `sourceObject` - the source object: `sourceListProperty` - a property on the source object that resolves to a `List`: `targetJTable` - the target `JTable` **Returns:**: the `JTableBinding` **Throws:**: `java.lang.IllegalArgumentException` - if `sourceListProperty` is `null`

---


### createJTableBinding

```
public static <E,SS> JTableBinding<E,SS,javax.swing.JTable> createJTableBinding(AutoBinding.UpdateStrategy strategy,
                                                                                SS sourceObject,
                                                                                Property<SS,java.util.List<E>> sourceListProperty,
                                                                                javax.swing.JTable targetJTable,
                                                                                java.lang.String name)
```

:   Creates a named `JTableBinding` from an object and property that resolves to a `List` and a direct reference to a `JTable`.

    :   **Parameters:**: `strategy` - the update strategy: `sourceObject` - the source object: `sourceListProperty` - a property on the source object that resolves to a `List`: `targetJTable` - the target `JTable` **Returns:**: the `JTableBinding` **Throws:**: `java.lang.IllegalArgumentException` - if `sourceListProperty` is `null`

---


### createJTableBinding

```
public static <E,TS> JTableBinding<E,java.util.List<E>,TS> createJTableBinding(AutoBinding.UpdateStrategy strategy,
                                                                               java.util.List<E> sourceList,
                                                                               TS targetObject,
                                                                               Property<TS,? extends javax.swing.JTable> targetJTableProperty)
```

:   Creates a `JTableBinding` from a direct reference to a `List` and an object and property that resolves to a `JTable`.

    :   **Parameters:**: `strategy` - the update strategy: `sourceList` - the source `List`: `targetObject` - the target object: `targetJTableProperty` - a property on the target object that resolves to a `JTable` **Returns:**: the `JTableBinding` **Throws:**: `java.lang.IllegalArgumentException` - if `targetJTableProperty` is `null`

---


### createJTableBinding

```
public static <E,TS> JTableBinding<E,java.util.List<E>,TS> createJTableBinding(AutoBinding.UpdateStrategy strategy,
                                                                               java.util.List<E> sourceList,
                                                                               TS targetObject,
                                                                               Property<TS,? extends javax.swing.JTable> targetJTableProperty,
                                                                               java.lang.String name)
```

:   Creates a named `JTableBinding` from a direct reference to a `List` and an object and property that resolves to a `JTable`.

    :   **Parameters:**: `strategy` - the update strategy: `sourceList` - the source `List`: `targetObject` - the target object: `targetJTableProperty` - a property on the target object that resolves to a `JTable` **Returns:**: the `JTableBinding` **Throws:**: `java.lang.IllegalArgumentException` - if `targetJTableProperty` is `null`

---


### createJTableBinding

```
public static <E,SS,TS> JTableBinding<E,SS,TS> createJTableBinding(AutoBinding.UpdateStrategy strategy,
                                                                   SS sourceObject,
                                                                   Property<SS,java.util.List<E>> sourceListProperty,
                                                                   TS targetObject,
                                                                   Property<TS,? extends javax.swing.JTable> targetJTableProperty)
```

:   Creates a `JTableBinding` from an object and property that resolves to a `List` and an object and property that resolves to a `JTable`.

    :   **Parameters:**: `strategy` - the update strategy: `sourceObject` - the source object: `sourceListProperty` - a property on the source object that resolves to a `List`: `targetObject` - the target object: `targetJTableProperty` - a property on the target object that resolves to a `JTable` **Returns:**: the `JTableBinding` **Throws:**: `java.lang.IllegalArgumentException` - if `sourceListProperty` or `targetJTableProperty` is `null`

---


### createJTableBinding

```
public static <E,SS,TS> JTableBinding<E,SS,TS> createJTableBinding(AutoBinding.UpdateStrategy strategy,
                                                                   SS sourceObject,
                                                                   Property<SS,java.util.List<E>> sourceListProperty,
                                                                   TS targetObject,
                                                                   Property<TS,? extends javax.swing.JTable> targetJTableProperty,
                                                                   java.lang.String name)
```

:   Creates a named `JTableBinding` from an object and property that resolves to a `List` and an object and property that resolves to a `JTable`.

    :   **Parameters:**: `strategy` - the update strategy: `sourceObject` - the source object: `sourceListProperty` - a property on the source object that resolves to a `List`: `targetObject` - the target object: `targetJTableProperty` - a property on the target object that resolves to a `JTable` **Returns:**: the `JTableBinding` **Throws:**: `java.lang.IllegalArgumentException` - if `sourceListProperty` or `targetJTableProperty` is `null`

---


### createJComboBoxBinding

```
public static <E> JComboBoxBinding<E,java.util.List<E>,javax.swing.JComboBox> createJComboBoxBinding(AutoBinding.UpdateStrategy strategy,
                                                                                                     java.util.List<E> sourceList,
                                                                                                     javax.swing.JComboBox targetJComboBox)
```

:   Creates a `JComboBoxBinding` from direct references to a `List` and `JComboBox`.

    :   **Parameters:**: `strategy` - the update strategy: `sourceList` - the source `List`: `targetJComboBox` - the target `JComboBox` **Returns:**: the `JComboBoxBinding`

---


### createJComboBoxBinding

```
public static <E> JComboBoxBinding<E,java.util.List<E>,javax.swing.JComboBox> createJComboBoxBinding(AutoBinding.UpdateStrategy strategy,
                                                                                                     java.util.List<E> sourceList,
                                                                                                     javax.swing.JComboBox targetJComboBox,
                                                                                                     java.lang.String name)
```

:   Creates a named `JComboBoxBinding` from direct references to a `List` and `JComboBox`.

    :   **Parameters:**: `strategy` - the update strategy: `sourceList` - the source `List`: `targetJComboBox` - the target `JComboBox` **Returns:**: the `JComboBoxBinding`

---


### createJComboBoxBinding

```
public static <E,SS> JComboBoxBinding<E,SS,javax.swing.JComboBox> createJComboBoxBinding(AutoBinding.UpdateStrategy strategy,
                                                                                         SS sourceObject,
                                                                                         Property<SS,java.util.List<E>> sourceListProperty,
                                                                                         javax.swing.JComboBox targetJComboBox)
```

:   Creates a `JComboBoxBinding` from an object and property that resolves to a `List` and a direct reference to a `JComboBox`.

    :   **Parameters:**: `strategy` - the update strategy: `sourceObject` - the source object: `sourceListProperty` - a property on the source object that resolves to a `List`: `targetJComboBox` - the target `JComboBox` **Returns:**: the `JComboBoxBinding` **Throws:**: `java.lang.IllegalArgumentException` - if `sourceListProperty` is `null`

---


### createJComboBoxBinding

```
public static <E,SS> JComboBoxBinding<E,SS,javax.swing.JComboBox> createJComboBoxBinding(AutoBinding.UpdateStrategy strategy,
                                                                                         SS sourceObject,
                                                                                         Property<SS,java.util.List<E>> sourceListProperty,
                                                                                         javax.swing.JComboBox targetJComboBox,
                                                                                         java.lang.String name)
```

:   Creates a named `JComboBoxBinding` from an object and property that resolves to a `List` and a direct reference to a `JComboBox`.

    :   **Parameters:**: `strategy` - the update strategy: `sourceObject` - the source object: `sourceListProperty` - a property on the source object that resolves to a `List`: `targetJComboBox` - the target `JComboBox` **Returns:**: the `JComboBoxBinding` **Throws:**: `java.lang.IllegalArgumentException` - if `sourceListProperty` is `null`

---


### createJComboBoxBinding

```
public static <E,TS> JComboBoxBinding<E,java.util.List<E>,TS> createJComboBoxBinding(AutoBinding.UpdateStrategy strategy,
                                                                                     java.util.List<E> sourceList,
                                                                                     TS targetObject,
                                                                                     Property<TS,? extends javax.swing.JComboBox> targetJComboBoxProperty)
```

:   Creates a `JComboBoxBinding` from a direct reference to a `List` and an object and property that resolves to a `JComboBox`.

    :   **Parameters:**: `strategy` - the update strategy: `sourceList` - the source `List`: `targetObject` - the target object: `targetJComboBoxProperty` - a property on the target object that resolves to a `JComboBox` **Returns:**: the `JComboBoxBinding` **Throws:**: `java.lang.IllegalArgumentException` - if `targetJComboBoxProperty` is `null`

---


### createJComboBoxBinding

```
public static <E,TS> JComboBoxBinding<E,java.util.List<E>,TS> createJComboBoxBinding(AutoBinding.UpdateStrategy strategy,
                                                                                     java.util.List<E> sourceList,
                                                                                     TS targetObject,
                                                                                     Property<TS,? extends javax.swing.JComboBox> targetJComboBoxProperty,
                                                                                     java.lang.String name)
```

:   Creates a named `JComboBoxBinding` from a direct reference to a `List` and an object and property that resolves to a `JComboBox`.

    :   **Parameters:**: `strategy` - the update strategy: `sourceList` - the source `List`: `targetObject` - the target object: `targetJComboBoxProperty` - a property on the target object that resolves to a `JComboBox` **Returns:**: the `JComboBoxBinding` **Throws:**: `java.lang.IllegalArgumentException` - if `targetJComboBoxProperty` is `null`

---


### createJComboBoxBinding

```
public static <E,SS,TS> JComboBoxBinding<E,SS,TS> createJComboBoxBinding(AutoBinding.UpdateStrategy strategy,
                                                                         SS sourceObject,
                                                                         Property<SS,java.util.List<E>> sourceListProperty,
                                                                         TS targetObject,
                                                                         Property<TS,? extends javax.swing.JComboBox> targetJComboBoxProperty)
```

:   Creates a `JComboBoxBinding` from an object and property that resolves to a `List` and an object and property that resolves to a `JComboBox`.

    :   **Parameters:**: `strategy` - the update strategy: `sourceObject` - the source object: `sourceListProperty` - a property on the source object that resolves to a `List`: `targetObject` - the target object: `targetJComboBoxProperty` - a property on the target object that resolves to a `JComboBox` **Returns:**: the `JComboBoxBinding` **Throws:**: `java.lang.IllegalArgumentException` - if `sourceListProperty` or `targetJComboBoxProperty` is `null`

---


### createJComboBoxBinding

```
public static <E,SS,TS> JComboBoxBinding<E,SS,TS> createJComboBoxBinding(AutoBinding.UpdateStrategy strategy,
                                                                         SS sourceObject,
                                                                         Property<SS,java.util.List<E>> sourceListProperty,
                                                                         TS targetObject,
                                                                         Property<TS,? extends javax.swing.JComboBox> targetJComboBoxProperty,
                                                                         java.lang.String name)
```

:   Creates a named `JComboBoxBinding` from an object and property that resolves to a `List` and an object and property that resolves to a `JComboBox`.

    :   **Parameters:**: `strategy` - the update strategy: `sourceObject` - the source object: `sourceListProperty` - a property on the source object that resolves to a `List`: `targetObject` - the target object: `targetJComboBoxProperty` - a property on the target object that resolves to a `JComboBox` **Returns:**: the `JComboBoxBinding` **Throws:**: `java.lang.IllegalArgumentException` - if `sourceListProperty` or `targetJComboBoxProperty` is `null`


---


|  |  |  |
| --- | --- | --- |
| **Overview** \| **Package** \| **Class** \| **Use** \| **Tree** \| **Deprecated** \| **Index** \| **Help** | |  |
| **PREV CLASS**   NEXT CLASS | **FRAMES**    **NO FRAMES**     **All Classes** |  |
| SUMMARY: NESTED \| FIELD \| CONSTR \| METHOD | DETAIL: FIELD \| CONSTR \| METHOD |  |


---
